# Anti-N-methyl-D-aspartate Receptor Autoimmune Encephalitis: A Case Report

**DOI:** 10.31729/jnma.7307

**Published:** 2022-11-30

**Authors:** Bikash Yadav, Dhiraj Chaurasia, Abhiyan Kharel, Krishna Dhungana

**Affiliations:** 1 Kathmandu Medical College and Teaching Hospital, Sinamangal, Kathmandu, Nepal; 2 Department of Neuromedicine, Kathmandu Medical College and Teaching Hospital, Sinamangal, Kathmandu, Nepal

**Keywords:** *case reports*, *encephalitis*, *immunotherapy*, *methylprednisolone*

## Abstract

Anti-N-methyl-D-aspartate receptor encephalitis is a form of autoimmune encephalitis with acute or subacute neuropsychiatric symptoms. Despite this fact, due to a couple of factors, this condition remains insufficiently acknowledged and is an under-recognised clinical scenario. We describe a case of a patient presenting with fever, headache and altered sensorium along with a history of disorientation, episodes of abnormal body movements and loss of consciousness in the later phase. She was initially thought to have Status epilepticus with tuberculous meningoencephalitis but her cognitive functions did not improve despite appropriate treatment. She displayed features away from the usual course of disease leading to suspicions of Autoimmune Encephalitis and Anti-N-methyl-D-aspartate receptor reports later confirmed the diagnosis. Methylprednisolone and Intravenous Immunoglobulin was started empirically and she was discharged in stable health with stabilised emotional and cognitive function with Azathioprine and Levetiracetam continued. Our findings suggested early diagnosis and prompt immunotherapy treatment beneficial for the outcome.

## INTRODUCTION

Anti-N-methyl D-aspartate receptor (anti-NMDAR) encephalitis is a variant of autoimmune encephalitis characterised by complex neuropsychiatric features and the presence of Immunoglobulin G (IgG) antibodies against the subunit of the NMDA receptor 1 (NR1) in the central nervous system (CNS).^1^ The syndrome is frequently associated with ovarian teratomas and women are disproportionately affected. The classic presentation involves a constellation of neuropsychiatric signs and symptoms, including memory loss, hallucinations and decreased level of consciousness.^2^ However, variations in clinical presentation and nonsequential multiphasic courses often lead to delays in diagnosis.^3^ Therefore, this condition remains under-recognized despite being increasingly evident in the literature.

## CASE REPORT

A 22-year-old female presented to the emergency department of Patan Hospital with chief complaints of fever for one and a half months, fainting, headache and altered sensorium for a month and abnormal body movement for two days. The fever was of low grade with a maximum temperature of 100°F associated with generalised weakness and loss of appetite. She also had a significant history of headaches with holocranial type of heaviness with no aura nor any typical pattern or any specific association. After a month of these symptoms, her family members gave a history of emotional instability with features such as being easily irritable, annoying, irrelevant talking, laughing and inappropriately smiling at times along with a decreased level of awareness of surroundings and non-communicative at times. These symptoms gradually progressed to slow mentation.

The patient was admitted and during her stay, for two weeks her symptoms further worsened. She became disoriented, stopped opening her eyes and at times remained staring with vacant looks. For this she was evaluated and referred to higher centres in Kathmandu and was treated on a line of tubercular meningoencephalitis and on the benefit of the doubt for viral, however, the patient showed little improvement. Her cerebrospinal fluid (CSF) analysis report shows a total count (TC) of 55/mm^3^ neutrophils of 16, lymphocytes of 85, protein of 133 mg/dl, lactate dehydrogenase (LDH) of 69 unit/dl, lactate of 4 mmol/lit, adenosine deaminase (ADA) of 17.9 U/litre. The CSF culture showed no growth of any organism, no detection of cryptococcal antigen, tuberculosis (TB) on gene Xpert, and Herpes Simplex Virus (HSV) on analysis.^1,2^

One day prior to admission to the hospital, she suddenly developed abnormal body movement in the form of uprolling of eyes, clenching of teeth, frothing from the mouth, stiffening of the upper and lower limb for a few minutes followed by postictal confusion. After two hour of the first episode, she had similar multiple episodes without regaining consciousness. Then she was referred to the neuro medicine department for further management.

She was diagnosed with status epilepticus with tubercular meningoencephalitis and treated in the same line with antiepileptics and category 1 antitubercular treatment. Her seizures during the hospital stay were controlled with two antiepileptics drugs but her cognitive function did not improve. She had features like catatonia, inappropriate smiling, muttering, orofacial and limb dyskinesia, and fluctuating Glasgow Coma Scale (GCS) and she was also evaluated by a psychiatrist for the opinion.

As it was not a usual course, the anti-NMDA receptor antibody test in repeated Lumbar puncture or autoimmune encephalitis was sent since the condition of the patient was not improving and a young female with such symptoms was strongly suggested for Autoimmune Encephalitis. She was started with methylprednisolone and intravenous immunoglobulin (IVIg) empirically for five days. However, her report was awaited. Investigations were done to understand the condition where Escherichia coli was isolated from Foley catheter tip culture and Klebsiella was isolated from blood culture.

**Figure 1:**
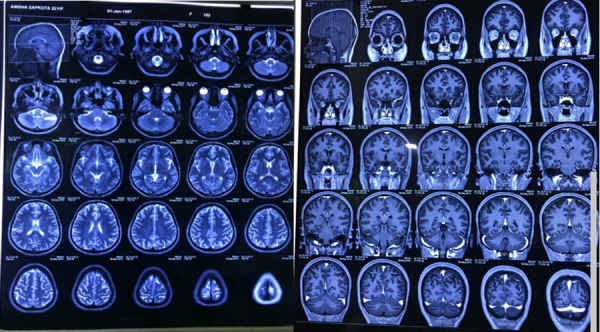
MRI study of the brain showing nonenhancing small high signal intensity in the right thalamus.

The sent anti NMDA antibody report came positive as well. The Magnetic Resonance Imaging (MRI) of the brain showed non-enhancing small high signal intensity in the right thalamus, otherwise, there was a normal MRI study of the brain (Figure 1).

The spinal cord was normal on whole spine screening. Screening of the abdomen showed no abnormalities in the liver, gallbladder (GB), spleen, kidney, pancreas, uterus and adnexa. After starting drug therapy, the patient then started to show some response on successive days. Her GCS was 15 and she had progressive stabilised emotional and cognitive function with no seizures and was discharged in stable health status. She has been given tablet Azathioprine 100 mg, and tablet Levera 1000 mg, both twice a day.

Due to some clinical manifestation, her ultrasonography report of the abdominal and pelvis revealed a small hyperechoic lesion in the right adnexal to a pouch of the Douglas region for which she underwent successful laparoscopic dermoid evacuation. Later, her drug doses were changed to Tablet Azathioprine 50 mg, and Tablet Levera 1000 mg twice daily. After a few months, the dosage of these drugs was tapered. She still complains of right-sided body and vision weakness, sometimes auto tongue twisting. Now she was a regular follow-up.

## DISCUSSION

Anti-NMDA receptor autoimmune encephalitis occurs when the component of the immune system called antibodies react against brain protein 'N-methyl-D-aspartate'. It manifests with characteristic features like initial headaches, low-grade fever, progressively prominent psychiatric symptoms, cognitive dysfunction like short-term memory loss and 1020 days later motor dysfunctions such as epileptic movements and dyskinetic movements, autonomic instability as hypoventilation, cardiac arrhythmia, and impaired consciousness.^4,5^ Oro-facial dyskinesias such as grimacing or lip smacking manifested may lead to misdiagnosing of seizures.^4^

Studies in the past have suggested that investigations such as Brain MRI, EEG and biopsy are usually nonspecific for diagnosis and anti-NMDAR antibodies in the CSF or serum thus serve as the benchmark for diagnosis in the current setting.^4,6^ After performing a series of investigations we went for testing anti-NMDA receptor antibodies in CSF, which was positive. It is reported that although there is a controversy between testing for serum or CSF antibody titres, CSF titres generally appear to correlate with disease activity.^7^ EEG supports diagnosing it because 90% of patients with anti-NMDAR antibodies show slow-moving rhythmic activities. After getting diagnosed with anti-NMDAR encephalitis, we should look for tumours inside patients' bodies as 5% of men and 20-30% of women of the age group 20-35 years have tumours inside them.^8^ In our patient, there was no tumour initially but found a dermoid cyst after a year on a USG scan which was surgically removed.

The treatment must cover both the cause and clinical consequences of encephalitis (inflammation, behaviour and psychotic symptoms).^4^ Generally treatment for autoimmune encephalitis is given prior to the test result which may include steroids and/or IVIG.^5^ For our patient we started her with methylprednisolone and IVIg empirically for 5 days after sending the anti-NMDAR antibody test. For the immediate management of behavioural and psychotic symptoms azathioprine 50mg twice a day and Levera 500 mg twice a day was started. Surgical removal of tumours such as ovarian teratoma if present, is needed to cure this clinical condition effectively.^4^ In this case ovarian teratoma was detected after a year and was removed by laparoscopic surgery at a higher health centre.

The initial clinical evaluations were suggestive of Status epilepticus with tuberculous meningoencephalitis, later investigations demonstrated evidence suggesting the actual disease changed the entire course of treatment for the patient. Overall it was a virtue of a timely diagnosis and intervention that saved the day.
